# Comparing public-health research priorities in Europe

**DOI:** 10.1186/1478-4505-7-17

**Published:** 2009-07-14

**Authors:** Mark McCarthy, Gabrielle Harvey, Claudia Conceição, Giuseppe la Torre, Gabriel Gulis

**Affiliations:** 1 Department of Epidemiology and Public Health, University College London, London, UK; 2 National School of Public Health, New University of Lisbon, Lisbon, Portugal; 3 Institute of Hygiene, Catholic University of the Sacred Heart, Rome, Italy; 4 Unit for Health Promotion Research, University of Southern Denmark, Esbjerg, Denmark

## Abstract

**Background:**

Despite improving trends, countries in Europe continue to face public-health challenges. This study investigated the priorities of stakeholders for research to meet these challenges.

**Methods:**

Public-health research includes population-level and health-system research, but not clinical or biomedical research. The study drew on data from three surveys undertaken through collaboration in *SPHERE *(Strengthening Public Health Research in Europe). There was participation of ministries in 18 of 28 (64% response) European countries, from 22 of 39 (56% response) member national associations of the European Public Health Association, and from 80 civil society health organisations (53% of members of the European Public Health Alliance)

**Results:**

Public-health research fields included disease control, health promotion and health services. Ministries of health, rather than ministries of science or education, mostly took responsibility for public-health research: they reported varied but well-defined areas for research in relation to national health plans and programmes. National public health associations reported research priorities across most fields of public health, although with some European regional differences. Civil society health organisations prioritised health promotion research nationally, but also health services research internationally. There was less research reported on methods, such as modelling and economic analysis, wider determinants of health, and public-health interventions.

**Conclusion:**

Systematic collaboration between stakeholders across European countries would enhance knowledge and promote innovation to address contemporary public-health challenges.

## Introduction

The contemporary goals for public health in Europe are to improve health through more effective programmes and to understand better the causes of continuing disease and disability [1]. Health in Europe is better than ever before, yet there remain substantial challenges of premature disease – with variations geographically, between social groups and for minorities – and care for an ageing population. And while cardiovascular disease, cancer and injuries are not overcome, new diseases of behaviour such as HIV/AIDS and obesity are arising. In response, health systems for prevention, treatment and care need to be improved, with an emphasis on effectiveness, efficiency and equity.

Public-health research is aims at improving the quality of life of citizens. 'Public health' has different translations and meanings in European languages and cultures [2], but indicates a population-level approach with a likelihood of society-wide benefits. Public-health research is not driven by the commercial considerations, but seeks to engage with public attitudes and motivations to achieve collective benefits. While governments disperse national resources for research in 'health' in general, our focus is on public-health research in particular.

Knowledge from research can be used in policy-making and practice; and equally users and producers of research can propose research priorities that will increase useable knowledge [3]. Europe makes a substantial contribution to world public-health research, contributing approximately one third of all output across different fields [4]. Yet European countries differ in their levels of support, their readiness to learn from each other, and their capacity to contribute to Europe-wide programmes. Understanding research priorities is the necessary basis for developing a strategy to strengthen public health, and thereby achieve better health in Europe.

The World Health Organisation Headquarters supports three international structures on health research. Over the past decade, the Global Forum for Health Research has held conferences to promote research in low and middle income countries [5], and the growth of international funding for research on HIV, malaria and tuberculosis, partly reflects successful messages from the Forum. Strengthening capacity of researchers in poorer countries for evidence-based policy and economic health research is led by the Alliance for Health Policy and Systems Research [6], while the Council on Health Research for Development [7] engages directly with governments and supports country partnerships. However, WHO European Region has not been strongly involved in this work, and has directed its attention towards evidence synthesis for policy questions [8].

Health research is supported by the European Union through the European Commission's Directorate for Research [9]; and the Directorate for Health [10], and other directorates for fields including telecommunications, food safety and environment, also support work relevant to public health. However, the European Commission's Sixth Framework Research Programme gave a strong priority to gene research and its biomedical applications in therapeutics, and its funding for health research was limited [11,12]. In response to a call by the European Commission for research on policy, *SPHERE *(Strengthening Public Health Research in Europe) was established to gather information and produce knowledge on the state of public health research in Europe [13].

*SPHERE *had 18 partners from 12 European countries, working with the European Public Health Association which is the membership organisation of national public health associations. There were two broad objectives – to describe public-health research literatures and to determine priorities for research among European stakeholders. A growing range and volume of research is presented at the annual meetings of the European Public Health Association [14], as well as in more specialised fields including quality of care [15], safety [16], epidemiology [17] and health technology assessment [18]. Bibliometric studies in *SPHERE *[19] showed substantial differences between countries, with the highest publications per capita from the Nordic countries and the lowest from the EU new member states [20]. Publications are in national, Europe-wide and international journals, and mainly in English [4,21]. The literatures were also investigated in six broad research themes – health management, health promotion, health services research, genetic epidemiology, infectious disease control and environmental health [22-27].

Five groups of stakeholders were included in *SPHERE*. Studies of the European institutions provided evidence on the historical development of public-health research in Europe [28]. Support for public-health research training was described through surveys of the main research institutions [29]. Studies were conducted separately on national ministries of health [30], national professional organizations, and national and European civil society organizations [31]. This paper draws on these three last studies to compare perspectives on priorities of these stakeholders for public-health research.

## Methods

To develop a European perspective on public-health research, *SPHERE *considered the research interests of the Sections of the European Public Health Association and topics at annual conferences [15], the Cochrane Public Health Group [32] and the European Commission's research programme [9]. A broad definition of public-health research was developed and agreed by partners in *SPHERE*: "Public-health research refers to the organized quest for new knowledge to protect, promote and improve people's health. It is undertaken at population or health services level, in contrast to laboratory (cellular) or clinical (individual) health research, and differs from public health practice (which also uses scientific methods), as it is designed to obtain generalisable knowledge rather than to address specific programmes for service delivery. It is usually goal-oriented, addressing questions of policy relevance, and may be published in either academic journals or reports. It uses a range of observational methods, including surveys, registers, data sets, case studies and statistical modelling, and draws on disciplines including epidemiology, sociology, psychology and economics, and interdisciplinary fields of environmental health, health promotion, disease prevention, health care management, health services research and health systems research". This definition was the basis for the exploratory bibliometric studies of European public-health literatures that formed the first component of *SPHERE *[13].

**Figure 1:**
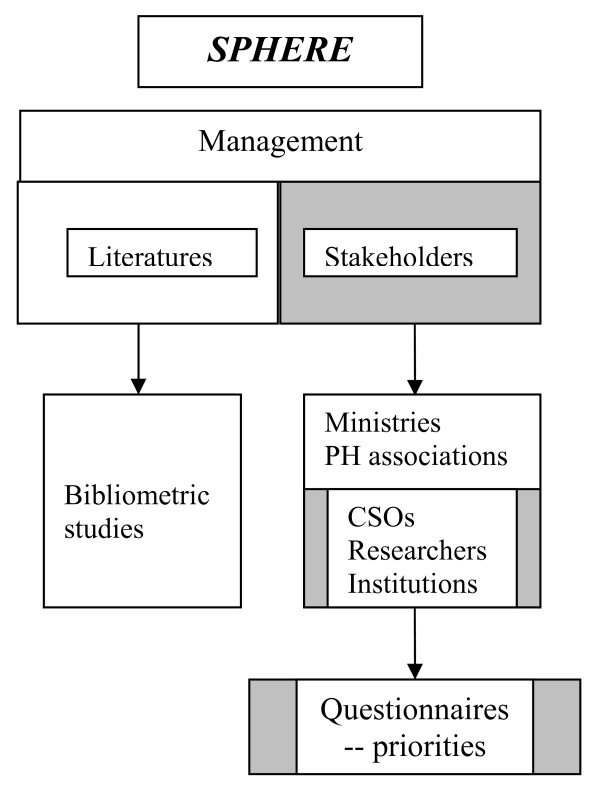
**Structure of study**. Questionniares were undertaken by three studies within SPHERE, and sections on priorities combined for analysis in this paper.

The three studies within *SPHERE *that contributed to this analysis of priorities [see Figure 1] were undertaken by separate partners. Each study had a similar objective, to describe public-health research from the perspective one group of these stakeholders, but the different range of topics was covered by the questionnaires in the three studies, drawing on the perspectives of contrasting groups of stakeholders across Europe – ministries of health, national public health associations and civil society organisations. In each study, the questionnaires were developed and reviewed between the *SPHERE *partners [see Additional Files 1]. Sampling frames were identified and e-mail and telephone contacts were made to identify suitable respondents.

With the completed questionnaires, data were extracted from forms. The sections on research priorities were brought together, and assessed using framework analysis [33]. Framework analysis was developed for use in applied policy research and provides a systematic way of analysing qualitative data. Steps include familiarization with the data, identification of a thematic framework, indexing, charting, and mapping; and interpretation [34]. For this study, responses were compared by questionnaire, country and research field.

### National Ministries of Health and Science

The study addressed Ministries of Health and the Ministries of Science of the (in 2005) 25 European Union countries, plus European Economic Area countries Iceland, Norway and Switzerland. Methods of identifying contacts in the 56 ministries included searching the Ministries' official websites, new persons proposed by other key persons within their Ministries, advice from *SPHERE *partners to help reach their ministries; and the national delegates of the European Medical Research Councils. A questionnaire broadly asking about support for public health research including a question on public health priorities: "What are the priority areas for public-health research in your country at present?" The questionnaire was sent to both ministries in each country, and followed up with repeated emails and phone calls to identify a respondent. The survey was done during the period January – October 2006, with follow-up to April 2007. Responses were gained from 41 out of 56 ministries, and from both or one ministry in 25 of the 28 countries (no response from either ministry in Luxembourg, Iceland or Poland). Sixteen ministries of health provided useable replies on priorities. Six ministries of education replied, of which two indicated the ministry of health as deciding priorities, two described biomedical priorities and two (Germany and Italy) ministries of education and research gave public-health research priorities rather than the Ministry of Health.

### National public health associations

The second study assessed perspectives of senior representatives from national public health associations about their perceived priorities for commissioning public-health research at national and local levels. Seeking to gain at least one response per country, the questionnaire, and follow-up emails, were sent in May-September 2006 to a total of 90 members of 39 national public health associations through the European Public Health Association. There were 23 response questionnaires analysed from 22 countries (country response rate 56%), including 13 from the 'old' (15) EU member states, 6 from the 'new' (12) member states and three from associated and neighbouring states. Respondents were asked to confirm public-health priorities from a list of 17 fields at national and international levels. The questionnaire answers were tabulated and analysed descriptively.

### Civil society health organisations

The third study was undertaken by a civil society organisation (SAVEZ) in an EU new member state, jointly with the European Public Health Alliance (EPHA), an umbrella group for health non-governmental organisations based in Brussels. A short questionnaire was sent by email to 150 member organisations within EPHA, to 47 members of the European Commission's European Health Policy Forum (with some overlap of the EPHA members) and also to a wider list of 1400 EPHA contacts across European countries. Responses were received from 80 civil society health organisations (CSHOs): half (53%) of the EPHA member organisations, and very few from other organisations. The responses covered 28 countries in total, with 31 from the 'old' (15 pre-2005) EU member states, 27 from the 'new' (12 post-2007) member states, and 13 from associated and neighbouring countries. About half of the CSHOs were working at international level, the rest were national or local. The CSHOs had a range in fields of interest within public health, most frequently in advocacy, health promotion, provision of services and training. In the questionnaire, respondents were asked 'Please describe what would be, from your perspective, the needs for public health research on national/regional and international level'. Answers from the questionnaires were tabulated and analysed descriptively.

## Results

### National ministries of health and science

The majority of countries did not formally have public-health research priorities, and some countries acknowledged that, "There are no ... priorities on public-health research as such", or "None has been explicitly publicised", while another respondent described priorities as "very broad". However, drawing from the information given by respondents and their direction towards web-sites, areas of public health research were described by at least one ministry for eighteen countries [see Additional Files 2].

**Figure 2:**
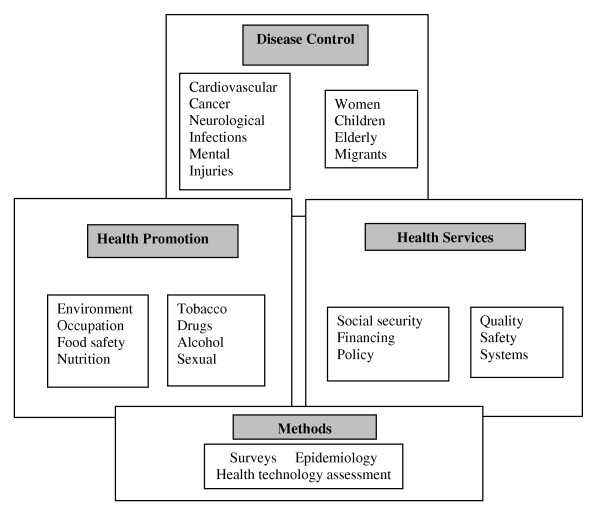
**Fields of public-health research**. Responses from the three questionnaires were considered within three practice fields, and methods research.

Ministries referred to thematic areas included in public health or health policy documents (including national health plans, public health plans) that identify needs and priorities in health research/public health research which can be summarised in three broad areas [see Figure 2]:

• Disease control: major diseases and target population groups

• Health promotion: environment and behaviours

• Health services: health systems and quality of services

There was also a theme on methods. Some ministries pointed to priorities as thematic areas included in national research plans, while and noted the research programmes that national agencies were leading or for which calls were open at the moment.

### National public health associations

National public health associations were asked their country's public-health research programmes using a checklist of potential research areas. Associations were asked to distinguish check public-health research priorities at national level and also those at international level. Table 1 shows that national associations identified quite strongly with most of the fields (migrant health was least noted).

**Table 1:** Public-health research priorities identified by respondents from national public health associations

		National N (%)	International N (%)
Disease control	Cardiovascular diseases	18 (78)	7 (30)

	Cancer	16 (70)	8 (35)

	Infectious diseases	17 (75)	7 (30)

	Mental health	15 (65)	-

	Migrant health	8 (35)	3 (13)

			

Health promotion	Environmental health	17 (74)	-

	Occupational medicine	15 (65)	-

	Bioterrorism	12 (53)	3 (13)

	Food safety and nutrition	18 (78)	

	Health education and promotion	15 (65)	5 (22)

	Drug addiction	13 (56)	6 (26)

			

Health services	Health services research	18 (78)	5 (22)

	Patient safety	15 (65)	-

	Health technology assessment	13 (56)	5 (22)

	Health management	14 (61)	4 (17)

	Use of medicines	13 (56)	4 (17)

			

Methods	Epidemiology	15 (65)	4 (17)

The most important priorities at international level for the Northern Europe group were: infectious diseases, cardiovascular diseases, cancer research with the same percentage (40%); for Southern Europe, health services, cancer research, drug addiction, food safety and nutrition, health technology assessment and mental health, with the same percentage (29%). Eastern European countries gave higher ratings to international research than the Northern and Southern groups: their highest priorities at international level were cardiovascular diseases and mental health (60%).

Dividing the countries in three geographical macro-areas, the most important priorities at national level for a Northern-Europe group (Austria, Belgium, France, Finland, Germany, Iceland, Ireland, the Netherlands, Norway, Sweden and UK) were health services and patient safety, with the same percentage (90%); for a Southern-Europe group (Albania, Greece, Italy, Malta, Portugal and Spain) the main priorities at national level were infectious diseases (86%), health services (71%) and cardiovascular diseases (71%); for all countries of Eastern Europe (Latvia, Lithuania, Poland, Romania and Slovakia) the main priorities at national level are food safety and nutrition, environmental health and occupational health.

### Civil society health organisations

Respondents to the civil society health organisations' questionnaire were asked to describe their perspective of the needs for public-health research, on national/regional and international level, with up to three themes. General areas – eg. public health, or environment and health – were most frequently proposed. In a middle range were topics linked to behavioural determinants of health, and there were also some 'organisational' items [see Table 2].

**Table 2:** Civil society health organisations: national and international level public-health research priorities

Public health research fields	Public health research topics	Prioritised – National	Prioritised – International
General	Public health, population health	11	9
			
Disease control	Sexual and reproductive health	2	3
	Mental health	3	5
	Cancer	4	2
	Ageing	4	4
			
Health promotion	Environment and health	6	5
	Economic, social determinants of health	4	2
	Obesity, nutrition	5	6
	Tobacco	4	5
	Injury prevention	3	2
	Lifestyle	0	4
	'Awareness' research	4	4
			
Health services	Health care systems, reforms, finances, access	2	6
	Smaller size research	3	4
	Cost effectiveness of prevention, treatment	1	4
	Implementation research	0	3
	Health and social care	0	3

Less frequently mentioned were specific diseases and conditions (meningitis, rheumatism, allergies, diabetes, rare diseases, HIV/AIDS, anaemia, asthma, violence, drug abuse), issues in behaviour (health education, community resilience), organisational aspects of health care (hospital care, palliative care, post-hospital and home care, quality improvement, private sector collaboration), information (national health surveys, DALYs and burden of disease, data collection systems), professional needs (medical education, nursing, midwifery) and four broader topics – rural health, support to South East Europe, scientific writing, and pharmaceutical products.

### Comparison between respondent groups

The questionnaires on priorities differed between the three surveys, which were undertaken by researchers working separately in three European countries, and the questionnaire design could have contributed to the differences in level of response by each group of respondents. The national public health associations were given the greatest number of fields, and they positively checked the majority of options. Ministries had a single open question: their range of responses was broader than for the national associations, but ministries varied considerably in the detail of their responses and fewer indicated as many fields as did the national public health associations. The civil society health organisations generally reported only one or two fields, relating to their own more specialised interests, and they gave least attention to the health services fields nationally although they did indicate support for these internationally.

The responses from the three sets of questionnaires covered three broad activities of public health research – disease control, health promotion, and health services – are set out schematically in Figure 2. Although research on diseases and on health behaviours formed the largest fields of the research priorities reported by ministries, these were not present in the responses of some ministries, and health services research and underlying methodologies were infrequently described. The disease categories reported by ministries included the common diseases – cancer, cardiovascular, diabetes, neurological, locomotor diseases, and (notably) several mentions of mental health but only one for injuries. Civil society health organisations identified more specific and rarer diseases, reflecting their greater interest in 'single issues'. Responses from the ministries [see Additional File 1] identified wider areas research – for example, technology assessment, pharmaceuticals safety, food and nutrition, health surveys and health systems – and also gave attention to population groups – women, children, older people and migrants. However, while social security and health inequalities were occasionally described by ministries, research for other wider determinants of health was rare.

## Discussion

This study is a first approach to describing the priorities of stakeholders for public-health research across Europe. Using questionnaires, with email and telephone contacts, responses were gained from ministries in eighteen countries, predominantly from the ministry of health, from national public health associations in 22 countries, and from 80 civil society health organisations with both national and international interests. Research priorities could be broadly described under headings of disease control, health promotion and health services, although open questions provided more varied responses. Although some ministries had defined research programmes, there did not appear to be either systematic collaboration or consensus on collective priorities to address European public health challenges. National public health associations supported research across a broad range of fields, while civil society health organisations reported priorities most frequently in health promotion, perhaps reflecting support for public-health activities of the Europe Commission's Directorate for Health [10].

The response rates for the target groups achieved in these studies were over 50%. For the ministries, the research team required persistence to identify a person who felt knowledgeable about their national situation and able to describe it in English. Research is not usually identified as a separate responsibility by ministries of health, while ministries of science tend to include public health within broader (and larger) health research programmes. The respondents on behalf of public health associations replied individually: in general, national associations do not have formal structures monitoring their country's research activities, although this is has now been proposed by EUPHA for future activity [35]. Civil society health organisations working internationally demonstrated interest in research issues and, reflecting the 'science in society' objectives of the European Commission [36], this is a potential strength for research in the future.

The study developed a definition of public-health research that included a broad range of fields at population level. WHO [37], describing 'health systems research' in low- and middle-income countries, identified 19 topics which overlap partly with our definition of public health research, while the Global Forum for Health Research uses the term 'research for health' to include health systems and clinical translational research. In the European Commission's Seventh Framework Programme [9], 'health research' has three pillars, approximately covering laboratory, clinical and public health: the public health section includes health systems research as well as health promotion, and supports organisational, social science and statistical studies. Although in some countries the term 'public health' has been reduced to being synonymous with health promotion, most European countries have a wider use to include health services research and disease control [2]. In our study, most ministries providing answers were able to identify national research programmes that related to the study definition of public-health research, but fewer countries had programmes specifically called public-health research. There is a need to develop consistent taxonomy, for public-health research based on stakeholders' perspective, to support both commissioning and exchange of knowledge in this field.

Our study showed that information on public-health research is not organised within countries in a systematic way. While ministries took a broad approach to public-health research fields, they more frequently identified research priorities in disease control and health behaviour, and less often health services, health systems and methodologies research. Moreover, there appear to be substantial differences between countries in interest in public-health research, which may reflect different cultures both for applied health research generally and also the level of development of 'new' public health practice, ie concerning chronic disease control through health promotion, health determinants and health systems rather than hygiene, screening and occupational disease.

Nor was not evident that these priorities were linked to the priorities either of the European Commission's Research Directorate or the European Region of the World Health Organisation. The European Regional Office of the World Health Organisation's 'Health 21' strategy in the 1990s required member states to support health research as one of their health goals [28]. However, WHO EURO did not press this requirement at national level, and WHO moved towards research synthesis.

There have been calls for greater attention to support for public-health research across Europe [11,12]. More generally, the European Commission is seeking to achieve a 'European Research Area', whereby researchers, research commissioners and research users (the 'research triangle') coordinate their activities to maximise effectiveness and international competitiveness [38]. A European Research Area for public health will depend on engagement and collaboration of a broad range of stakeholders, including ministries of health, researchers and civil society organisations, while funding should also be sought from a wider range of sources that may include charities, insurance funds, and (for the new member states) EU regional funds. As current public health research output varies widely between countries, combining forces to address major public health research issues and develop knowledge for policy and practice will bring benefit to a larger proportion of Europe's citizens.

## Conclusion

This paper contributes to the information base to strengthen public health research in Europe and thereby address contemporary public health challenges. Public health research priorities in Europe were described by stakeholders across Europe through targeted questionnaires and responses were gained from most countries. Public-health research priorities are seen as the responsibility of ministries of health, in contrast with the priority for biomedical research of ministries of science, but health ministries have very varied conceptions of the research that is prioritised. In the future, a European public-health research area should develop priorities through collaboration between users and advocates, with research funded by a range of public bodies, and researchers providing the critical evidence of effectiveness – or otherwise – of organisational and population-level interventions.

## Competing interests

The authors declare that they have no competing interests.

## Authors' contributions

MM and GH coordinated the studies for *SPHERE*, CC undertook the study of national ministries of health and science, GT from the study of national public health associations and GG the study of civil society health organisations. Each study lead wrote their report for *SPHERE*, and MM and GC wrote the paper with all co-authors.

## Supplementary Material

Additional file 1 **Survey instruments in *SPHERE *surveys**. Provides the full questionnaires used by the three partners in the study. Click here for file

Additional file 2 **Countries' long-term national strategies for health research**. Listing of health research priorities indicated by ministries of health and of science in 18 European countries. Click here for file
