# Supplementary material for: Comparing public-health research priorities in Europe
Source: Health Res Policy Syst. 2009 Jul 14;7:17. doi: 10.1186/1478-4505-7-17 (PMC2732621; doi:10.1186/1478-4505-7-17)
Supplement: Additional file 1 — Survey instruments in SPHERE surveys. Provides the full questionnaires used by the three partners in the study. [file 1478-4505-7-17-S1.doc]

Survey instruments in SPHERE surveys

1. National ministries

***SPHERE***

Strengthening Public Health Research in Europe

<http://www.ucl.ac.uk/public-health/sphere/spherehome.htm>

[Name of the ministry – name of the country]

**strengthening public health research in europe**

**January 2006**

**Dear Sir/Madam,**

The European Public Health Association (EUPHA), through the Faculty of Public Health, University College London, United Kingdom, is undertaking a project, SPHERE (Strengthening Public Health Research in Europe), that will describe public health research at the European level, including support by national governments, and advise how it can be strengthened and most effectively integrated with European health policy.

The study will be undertaken by a consortium of European public health experts from EU Member States. We are asking your collaboration for a part of the study that is undertaken by the National School of Public Health, Lisbon, Portugal. The main objectives are to obtain information from Ministries of Health and of Science and Education about the support for public health research, evidence of success in knowledge development, use in the field and uptake by policy makers. We have designed a questionnaire to fulfil this objective, and are kindly asking your cooperation in completing it.

The coordinators of the project will send the first report to national governments for consultation, and we would be most grateful for your Ministry/Department’s involvement.

Despite consulting with partners and conducting internet searches, we remain unsure about some of the e - mail addresses for the International Department of the Ministry. If there is a department more oriented to international contacts than yourselves, we would be most grateful if you could pass their e - mail address to us.

The SPHERE project encompasses a large range of public health issues, and as such, other public health research and training institutions within Europe will also be approached to participate through the completion of questionnaires related to these topics.

We thank you for your participation in this project.

Yours faithfully,

Mark McCarthy

Professor of Public Health, University College London, United Kingdom

***SPHERE***

Strengthening Public Health Research in Europe

| Ministry of [one questionnaire for each ministry]  E - mail: |
| --- |

***Before completing this questionnaire please note the following:***

1 - **Public health research** usually refers to the organized quest for new knowledge to protect, promote and improve people’s health. As public health research can have some different understandings we would like to strength that in this study we are referring public health research:

i) that is undertaken at **population or health services level**, in contrast to laboratory (cellular) or clinical (individual) health research;

ii) that differs from public health practice (which also uses scientific methods), as it is **designed to obtain generalisable knowledge** rather than to address specific programmes for service delivery;

iii) that is usually goal-oriented, addressing questions of policy relevance, and may be published in either academic journals or as reports;

iv) that uses a range of observational methods, including surveys, registers, data sets, case studies and statistical modelling, and draws on disciplines including epidemiology, sociology, psychology and economics, and interdisciplinary fields of environmental health, health promotion, disease prevention, health care management, health services research and health systems research.

2 – Where an official document exists on the topics covered, we would be most appreciative if you could please attach them to the questionnaire, either by e - mail, fax, post or simply by including the web link. We would prefer English versions where possible, however, native language documents will also be accepted.

3 – We are sending the questionnaire as [name of the document].doc and PDF. We suggest you to save the file [name of the document].doc in your own computer as [name of the document]_anwer.doc before filling in the questionnaire.

***Part 1 – Personal particulars of respondent***

| Surname: | | Title: | | Initials: | |
| --- | --- | --- | --- | --- | --- |
| First name: | | | | | |
| Position in the Ministry/Department: | | | | | |
| Telephone and fax numbers: | | Country code: | | Area code: | |
| Tel (1) | Tel (2) | | Fax (1) | | Fax (2) |
| e-mail (1) | | | e-mail (2) | | |
| Other information that may help us to contact you: | | | | | |
| If we need to contact you for clarifications, and you are not available, who else can we contact? | | | | | |
| Surname: | | Title: | | Initials: | |
| First name: | | | | | |
| Telephone and fax numbers: | | Country code: | | Area code: | |
| Tel (1) | Tel (2) | | Fax (1) | | Fax (2) |
| e-mail (1) | | | e-mail (2) | | |
| Other information that may help us to contact him/her: | | | | | |

***Part 2 – Ministry support for public health research***

***2.1 - Identification of priorities and criteria for funds allocation.***

| 2.1.**1** - Please name the advisory structures or other organizations that are involved in the process of identification of **public health research** priorities in your country. | | |
| --- | --- | --- |
|  | | |
| 2.1.**2** - Succinctly describe the process of identification of **public health research** priorities in your country. | | |
|  | | |
| 2.1.**3** - What are the priority areas for **public health research** in your country at present? | | |
|  | | |
| 2.1.**4** - Is there an official document on the subject of priorities in public health research?  *Mark the appropriate answer with an X.* | Yes | No |
| If yes, please e - mail, fax or indicate its web address. | | |

| 2.1.**5** - What criteria does your Ministry/Department use to allocate funds for **public health research**? | | |
| --- | --- | --- |
|  | | |
| 2.1.**6** - Which organizations are involved in deciding which criteria will be used for the allocation of funds for **public health research**? | | |
|  | | |
| 2.1.**7** - Is there an official document on the subject of criteria for fund allocation?  *Mark the appropriate answer with an X.* | Yes | No |
| If yes, please e - mail, fax or indicate its web address. | | |

| 2.1.**8** – Briefly describe the process of fund allocation from your Ministry/Department to **public health research**. | | |
| --- | --- | --- |
|  | | |
| 2.1.**9** – How frequently does fund allocation occur? | | |
|  | | |
| 2.1.**10** - For how long are funds allocated? | | |
|  | | |
| 2.1.**11** - Is there an official document on the subject of process of fund allocation?  *Mark the appropriate answer with an X.* | Yes | No |
| If yes, please e - mail, fax or indicate its web address. | | |

| 2.1.**12** – What kind of **public health research** does your Ministry/Department fund?  *Mark the appropriate option with an X.* | |
| --- | --- |
| 1 – The Ministry/Department funds only internal **public health research** (on its own account) |  |
| 2 – The Ministry/Department contracts out all **public health research** |  |
| 3 – The Ministry/Department funds both internal and external (contracted out) public health research.  Please specify: |  |
| proportion of Ministry/Department budget (2006) for internal **public health research** | … % |
| proportion of Ministry/Department budget (2006) for contracted out **public health research** | … % |

| 2.1.**13** - Are funds allocated to **public health research** clearly identifiable in the Ministry/Department budget for 2006?  *Mark all the appropriate options with an X.* | |
| --- | --- |
| Yes |  |
| If so, please specify the % of total budget for research in your Ministry/Department | …% |
| No |  |
| Funds are available for **public health research**, but mixed in with other funding and hard or impossible to link explicitly with public health research |  |
| Other |  |
| Please, specify | |

***2.2 - Relationship between national health plans and criteria of research financed***

| 2.2.**14** - Are the criteria of **public health research** funding related to the national health plan? *Mark* *the appropriate option with an X.* | |
| --- | --- |
| We don't have a national health plan |  |
| Yes |  |
| Please specify in which way: | |
| If yes, please e - mail, fax or indicate the national health plan web address. | |
| No |  |

***2.3 - Evidence of success in development of knowledge***

| 2.3.**15** - Briefly describe the process of evaluation of **public health research** funded by your Ministry/Department. | | |
| --- | --- | --- |
|  | | |
| 2.3.**16** - Briefly describe how your Ministry/Department supports the communication/dissemination of results of **public health research**. | | |
|  | | |
| 2.3.**17** - Is there an official document on the subject of evaluation of success in development of knowledge on public health?  *Mark the appropriate answer with an X.* | Yes | No |
| If yes, please e - mail, fax or indicate its web address. | | |

***2.4 - Uses of the results of research in the field***

| 2.4.**18** - How does your Ministry/Department get information on the application of **public health research** findings in the field, by public health practitioners? | | | |
| --- | --- | --- | --- |
|  | | | |
| 2.4.**19** - Is there a system tracing the implementation of **public health research** data?  *Mark the appropriate option with an X.* | | | |
| Yes | | |  |
| Please describe | | | |
| No | |  | |
| 2.4.**20** - Is there an official document on the subject of the implementation of research findings in the field?  *Mark the appropriate answer with an X.* | Yes | No | |
| If yes, please e - mail, fax or indicate its web address. | | | |

***2.5 - Uptake of the results of research by policy makers***

| 2.5.**21** - What mechanisms or processes allow your policy makers to uptake the results of **public health research**? (please provide examples) | | |
| --- | --- | --- |
| Processes of the initiative and/or responsibility of your Ministry/ Department.  Other processes: | | |
| 2.5.**22** - Is there an official document on the subject of uptake of research results from policy makers?  *Mark the appropriate answer with an X.* | Yes | No |
| If yes, please e - mail, fax or indicate its web address. | | |

***Part 3 – Relationship between research practitioners and research funders***

| 3.**23** - Please provide the organizational reference for institutions/agencies that fund **public health research** (national or regional, public or private). | | |
| --- | --- | --- |
|  | | |
| 3.**24** - Briefly describe the process of coordination (between needs identified by users, scientific community and policy makers and resources for research) of public health research? | | |
|  | | |
| 3.**25** - Is there an official document on the subject of coordination of public health research?  *Mark the appropriate answer with an X.* | Yes | No |
| If yes, please e - mail, fax or indicate its web address. | | |

| 3.**26** - Is there a national board for research?  *Mark the appropriate answer with an X.* | |
| --- | --- |
| Yes |  |
| Please provide a contact and/or the web address of the board | |
| No |  |
| 3.**27** - Is there a national board for **public health research**?  *Mark the appropriate answer with an X.* | |
| Yes |  |
| Please provide contact of the board | |
| No |  |

***Part 4 – Progress of each country in commenting on, and supporting, the Research Directorate-General 7^th^*** Framework Programme.

| 4. **28** - Please send or indicate the web address where to find the most recent contribution from your government to the DG Research 7^th^ Framework Programme. |
| --- |
|  |
| 4.**29** - If not, please explain the process and national contribution to the DG Research 7^th^ programme. |
|  |

Part 5 –Strengthening of public health research at national level

| This project will integrate different parts to finally advise how public health research can be strengthened and most effectively integrated with European health policy. | | |
| --- | --- | --- |
| 5. **30** – What could be done, at national level, to develop public health research? | | |
|  | | |
| 5.**31** - Is there an official document on the subject of strengthening public health research at national level?  *Mark the appropriate answer with an X.* | Yes | No |
| If yes, please e - mail, fax or indicate its web address. | | |
| 5. **32** - In what ways could the European Union contribute to develop public health research in your country? | | |
|  | | |
| 5.**33** - Is there an official document on the subject of the contribution of the EU to the development of public health research in your country?  *Mark the appropriate answer with an X.* | Yes | No |
| If yes, please e - mail, fax or indicate its web address. | | |

***Questionnaire used for the 18 countries (15 former EU, Norway, Switzerland and Iceland)***

**Thank you very much** for answering this questionnaire. Should you have further queries, please contact Claudia Conceição or Patricia Barbosa at the addresses below. You can find more information on the project on the website:

<http://www.ucl.ac.uk/public-health/sphere/spherehome.htm>.

We would appreciate if you could send the completed questionnaire by e - mail to: [claudiac@ensp.unl.pt](mailto:claudiac@ensp.unl.pt) or by fax to FAX number + 351 217582754 by January 29^th^ 2006.

Contacts:

| Dr.ª Claudia Conceição  Tel + 351 217512100; +351 217512160  E-mail: [claudiac@ensp.unl.pt](mailto:claudiac@ensp.unl.pt) | Dr.ª Patricia Barbosa  Tel + 351 217512100; +351 217512156  E-mail: [patbarbosa@ensp.unl.pt](mailto:patbarbosa@ensp.unl.pt) |
| --- | --- |

Address:

Drª. Claudia Conceição

Escola Nacional de Saúde Pública

Avenida Padre Cruz

1600-560 Lisboa

Portugal

***Questionnaire used for the 10 new EU countries***

**Thank you very much** for answering this questionnaire. If you have any questions, please contact Ruta Taraseviciute at the address below. You can find more information on the project on the website: <http://www.ucl.uk/public-health/sphere/spherehome.htm>

You can find the electronic version of the questionnaire on the website of Kaunas Public Health Center: <http://www.kvsc.lt/english/index.htm>

We would appreciate if you could send the filled questionnaire by e-mail, fax or post till June 9, 2006 to:

Ruta Taraseviciute

Kaunas Public Health Center

K. Petrausko 24, Lt- 44156 Kaunas

Lithuania

Tel.: +370 7 331688

Fax.: +370 7 331680

E-mail: [ruta.taraseviciute@kvsc.lt](mailto:ruta.taraseviciute@kvsc.lt)

[end]

2 – National Public Health Associations

Dear Sir / Madam,

The European Public Health Association (EUPHA), through the Faculty of Public Health, United Kingdom, has proposed a project SPHERE (Strengthening Public Health in the European Region) that will describe public health research at the European level. More information about the project can be found from the attached covering letter and from the web-site <http://www.ucl.ac.uk/public-health/sphere/spherehome.htm>.

We kindly ask you to read the attached letter and to fill in the attached questionnaire and return it in two weeks (23th February 2006) and send it to [alice.mannocci@rm.unicatt.it](mailto:alice.mannocci@rm.unicatt.it). If there is another organization better oriented to fill in the questionnaire, please give us their contact information or simply forward the request, but please inform us about this.

Thanking You in anticipation,

Dr. Giuseppe La Torre

Dr. Alice Mannocci

Institute of Hygiene (Dir. Prof. G. Ricciardi),

Catholic University of the Sacred Heart, Rome

Largo Francesco Vito 1

00168 Rome

Italy
Email: [giuseppe.latorre@rm.unicatt.it](mailto:ly.vetik@tai.ee)

SPHERE – QUESTIONNAIRE on Public Health Research Funding in Europe (Work Package 4)

Country ..............................................................................

Name of the partner .............................................................................................

Institution ............................................................................................................

#### Section 1- Current methods on commissioning PH research at national and local levels

In your experience Periodical funding for PH research programs by national agencies is provided by:

<> Ministry of Health

<> Ministry of Scientific Research and/or Science and/or University

<> National Institute of Health

<> Ministry of Welfare

<> National Research Council

<> Regional Research Council/institute

<> Municipality

<> Min. of University

<> Industries

<> Foundations

<> Other (please specify)

................................................................................................................................................................................................................................................................................................................................

In your Region, Research projects are commissioned by

<> Regional Ministry of Health

<> Public Health Agency

<> Local Health Authority

<> Other (please specify)

..............................................................................................................................................................................................................................................................................................................

The following are some of Public Health priorities at national and international levels. Please fill with a tick and provide if possible a ID contact e-mail.

|  | National level | International level | Contact at national level |
| --- | --- | --- | --- |
| health services |  |  |  |
| infectious disease control |  |  |  |
| epidemiology |  |  |  |
| health management |  |  |  |
| cardiovascular disease research |  |  |  |
| cancer research |  |  |  |
| health education and promotion |  |  |  |
| use of medicines |  |  |  |
| drug addiction |  |  |  |
| food safety and nutrition |  |  |  |
| health technology assessment |  |  |  |
| migrant health |  |  |  |
| bio-terrorism |  |  |  |
| environmental health |  |  |  |
| mental health |  |  |  |
| patient safety |  |  |  |
| occupational health |  |  |  |

<> Other (please specify)

....................................................................................................................................................................................................................................................................................

#### Section 2 – Personal experience of research on Public Health practice

Please indicate up to 5 examples of publication (paper, report, book or chapter, etc.) of research findings in PH in the last five years

1)................................................................................................................................. ....................................................................................................................................

....................................................................................................................................

2)................................................................................................................................. ....................................................................................................................................

....................................................................................................................................

3)................................................................................................................................. ....................................................................................................................................

....................................................................................................................................

4)................................................................................................................................. ....................................................................................................................................

....................................................................................................................................

5)................................................................................................................................. ....................................................................................................................................

Has your own Institute a website where research findings on Public Health are shown?

<> Yes

<> No

<> No, but it will be implemented in a short period (within 3 months)

Has your own Institute a newsletter where research findings on Public Health are published?

<> Yes

<> No

<> No, but it will be implemented in a short period (within 3 months)

Please indicate (mark with a tick) if, in your experience, the following are barriers to undertaking better research in Public Health

|  | Strongly agree | Agree | Uncertain | Disagree |
| --- | --- | --- | --- | --- |
| Lack of Infrastructure |  |  |  |  |
| Lack of technological equipment |  |  |  |  |
| Lack of Research personnel |  |  |  |  |
| Unsatisfactory administrative personnel |  |  |  |  |
| Difficulties of publishing results of research |  |  |  |  |
| No interactions between policy makers’ needs and scientific needs |  |  |  |  |
| No Evidence on implementation of research findings in PH practice |  |  |  |  |

<> Other (please specify)

....................................................................................................................................................................................................................................................................................

#### Section 3 - Evidence on implementation of research findings in PH practice

How do you rate the implementation of your own research activities in Public Health practice?

<> Very high

<> High

<> Medium

<> Sufficient

<> Insufficient

How do you rate the activities of your National Society of PH in influencing PH practice?

<> Very high

<> High

<> Medium

<> Sufficient

<> Insufficient

Do you know about implementation of research findings in PH practice in your Country?

<> Yes

<> No

Is there a national database of research findings on Public Health?

<> Yes

<> No

<> No, but it will be implemented in a short period (within 3 months)

Please indicate up to 5 studies or reports on the work of influencing PH practice or on the “translation” of PH research in practice

1)................................................................................................................................. ....................................................................................................................................

....................................................................................................................................

2)................................................................................................................................. ....................................................................................................................................

....................................................................................................................................

3)................................................................................................................................. ....................................................................................................................................

....................................................................................................................................

4)................................................................................................................................. ....................................................................................................................................

....................................................................................................................................

5)................................................................................................................................. ....................................................................................................................................

....................................................................................................................................

#### Section 4 – Financial aspects

Please indicate the annual budget (in Euro) of your Country for Public Health Research

...............................................................................

How do you rate the annual budget for Public Health research at the National level?

<> Very high

<> High

<> Medium

<> Sufficient

<> Insufficient

Please indicate the annual budget (in Euro) of your Region for Public Health Research

...............................................................................

How do you rate the annual budget for Public Health research at the Regional level?

<> Very high

<> High

<> Medium

<> Sufficient

<> Insufficient

Please indicate the annual budget (in Euro) of Private Institutions in your Country for Public Health Research

...............................................................................

How do you rate the annual budget for Public Health research of Private Institutions at the National level?

<> Very high

<> High

<> Medium

<> Sufficient

<> Insufficient

Which are the three priorities areas that you would like to see future public health research directed towards?

........................................................................................................

........................................................................................................

.......................................................................................................

Thank you

[end]

Appendix 3 – Civil Society Health Organisations

SPHERE

Strengthening public health research in Europe

Questionnaire on Public Perception

February 2006

Dear Sir/Madam,

The European Public Health Association (EUPHA), through the Faculty of Public Health, (United Kingdom), has proposed a project SPHERE (Strengthening Public Health in the European Region) that will describe public health research at the European level, including support by national governments, and advise how it can be strengthened and most effectively integrated into European health policy.

The project is financially supported by the European Commission, DG Research

You can get more information about the project at its website

<http://www.ucl.ac.uk/public-health/sphere/spherehome.htm>

At present on level of the European Union there are two major research funding schemes in Europe. The “Framework 6” program directed by the Research and Development Directorate of the European Commission aims to support basic research and establish a European Integrated Research Area. Public health topics are included in this major research program and if not yet familiar, you can find more information about it at the following web site:

<http://fp6.cordis.lu/index.cfm?fuseaction=UserSite.FP6HomePage>

The second major public health resource under European Commission is the public health program of the EC directed by Directorate of public health and consumer protection. This program aims to fund more applied research, project dealing with implementation of new knowledge into practice. You can get more information about this program at the following website:

<http://europa.eu.int/comm/health/ph_programme/programme_en.htm>

The SPHERE study will be undertaken by a consortium of European public health experts from several EU Member States. We would like to ask for your collaboration to a part of the study that is undertaken by European Public Health Alliance (EPHA) and the Slovak Public Health Association (SAVEZ). Its objective is to obtain information from non-governmental organizations as representatives of public about public health research in Europe. We would be grateful if you could fill in the attached questionnaire and return it up to February 20, 2006 to Gabriel Gulis by E-mail ([ggulis@health.sdu.dk](mailto:ggulis@health.sdu.dk)).

Thank you for your participation!

Best wishes,

Gabriel Gulis Roxana Radulescu

SAVEZ EPHA

Name and address of your NGO

- 1. Area of work NGO
  2. Duration of existence
  3. Experience with international work (collaboration with international organizations, partnership in international projects etc.)
  4. Experience with international public health research work (public health research in this sense is considered as a non-clinical medicine research tackling major problems of population health. The above mentioned research programs describe more in depth what could be considered as public health research)

Awareness about international public health research possibilities before this questionnaire

- 1. Have you ever heard before receiving this questionnaire about Framework 6 and the Public Health Program of the EU? Please describe.

…………………………………………………………………………………………

- 1. Have you ever been asked to participate in an international project funded by major EU programs in field of public health? Please describe.
  2. Have you ever heard or been contacted in relation to other international public health research funding resources? Please describe

Experience with national, regional and international public health research

a. Have you ever been a partner within a national or regional public health research project? Please describe

b. Have you ever been a partner within an international public health research project? Please describe.

Please describe what would be, from your perspective, the needs for public health research on national/regional and international level

- 1. National and/or Regional

- 1. International

Thank you for your time and willingness to collaborate!

[end]
