# Supplementary material for: Comparing public-health research priorities in Europe
Source: Health Res Policy Syst. 2009 Jul 14;7:17. doi: 10.1186/1478-4505-7-17 (PMC2732621; doi:10.1186/1478-4505-7-17)
Supplement: Additional file 2 — Countries' long-term national strategies for health research. Listing of health research priorities indicated by ministries of health and of science in 18 European countries. [file 1478-4505-7-17-S2.doc]

*Additional File 2 – Countries’ long-term national strategies for public-health research*

Survey of National Ministries in European countries – Responses on current public-health research priorities

( - = no response received)

| **Austria** | **Federal Ministry for Health and Women** | **Federal Ministry for Education, Science and Culture** |
| --- | --- | --- |
|  | Health promotion with focus on woman’s and mental health, HIV, alcohol and tobacco, quality assurance. | - |
| **Belgium** | **Federal Public Service Health, Food Chain Safety and Environment** | **Federal Public Service Health, Food Chain Safety and Environment** |
|  | Mental Health: homeless persons, reorganisation of mental health care services; suicide  Drugs: research on the use and effects of drugs, double-diagnosis, ethnic minority drug abusers, effectiveness and efficacy of health education for benzodiazepines, crisis intervention  National Food consumption Survey  Research on the effects of tobacco | - |
| **Czech Republic** | **Ministry of Health** | **Ministry of Education, Youth and Sports** |
|  | Health status determinants;  Epidemiology of the selected diseases  Health promotion and disease prevention - health related behaviour of the Czech population | - |
| **Denmark** | **Ministry of the Interior and Health** | **Ministry of Science, Technology and Innovation** |
|  | Interdisciplinary research programme on the correlation between food, nutrition and health. | - |
| **Estonia** | **Ministry of Social Affairs** | **Ministry of Education and Research** |
|  | Health promotion  Infectious disease inc HIV/AIDS  Illegal drug consumption | - |
| **Finland** | **Ministry of Social Affairs and Health** | **Ministry of Education and Science** |
|  | Strategies for Social Protection 2010   - promoting health and functional capacity - making work more attractive - preventing and combating social exclusion - providing efficient services and income security | - |
| **France** | **Ministry of Health and Solidarity** | **Ministry of Higher Education and Research** |
|  | The main 2005-2006 calls for research projects in public health are about  • Relations between health and environment and health and occupation (National Research Agency)  • Social inequalities and health (IVRSP 2005)  • Development of new methodologies for evaluation of public health actions (IVRSP 2006).  The French national institute of health and medical research (INSERM) promotes the development of cohort studies. | - |
| **Germany** | **Federal Ministry of Health** | **Federal Ministry of Education and Research** |
|  | - | Health services/health care research  Research on Prevention and Health Promotion  Nursing Research |
| **Italy** | **Department of Education and Science** | **Ministry of Higher Education and Research** |
|  | 1. Health in the first period of lifetime, babyhood and adolescence 2. Great pathologies: cancer, cardiovascular diseases, diabetes and respiratory illnesses 3. Elderly and disabled 4. Mental health safeguard 5. Dependences to particular style of life 6. Families support 7. Immigrants and marginal social level health care 8. Follow-up of the diffusive illnesses 9. Food safety and nutrition 10. Veterinary health 11. Safeguard of the health and safety in the job places   12. Environment health | Thematic areas of the National Committee of Research  1. Oncology  2. Cardiovascular disease  3. Diabetes Type II – obesity  4. Neurodegenerative disease  5 Infectious disease  6. Stem cells  7. Permanent care  8. Social medical service link  9. Veterinary area (animal health – food safety) |
| **Lithuania** | **Ministry of Health** | **Ministry of Education and Science** |
|  | Prevention of cardiovascular diseases  Accessibility and availability of health care services  Prevention of cardiovascular diseases  Prevention of traumas and injuries | - |
| **Malta** | **Ministry of Health, the Elderly and Community Care** | **Ministry of Education** |
|  | Obesity, mental health and diabetes are deemed priority areas. | - |
| **The Netherlands** | **Ministry of Health, Welfare and Sport** | **Ministry of Education, Culture and Science** |
|  | “Preventie nota 2002-2006” addresses smoking, overweight, diabetes, alcohol prevention, mental diseases / depression, and counts as 6 priority diseases: Heart- & vessel diseases; cancer; COPD; diabetes mellitus; mental diseases (depression); failure of the locomotor apparatus.  In the decision making process on fund allocation, for instance in the ‘letters of assignment’ by the ministry of VWS to the ZonMW and RIVM, will refer to this ‘document on prevention’. | The Ministry of Education, Culture, and Science coordinates science and research in general. Priorities for Public Health Research specifically are set by the Ministry of Health, Welfare and Sport. |
| **Norway** | **Ministry of Health and Care Services** | **Ministry of Education and Research** |
|  | - Stem cells - Older people - Research on women illness - Mental health | - |
| **Portugal** | **Ministry of Health** | **Ministry of Science, Technology and Higher Education** |
|  | Priorities in 6 main areas (in the national health plan):   - Chronic diseases research (cardiovascular disease, cancer, HIV, diabetes, osteoarthritis, neurodegenerative disease) - Transmissible diseases (molecular epidemiology of transmissible diseases, tuberculosis, malaria, nosocomial infection) - Health technologies ( new diagnosis techniques, technologies assessment, increase of efficiency of technologies) - Health services research ( strategic analyze of health system evolution, knowledge of many areas of health services) - Health policy research (effectiveness of health programmes) - Migrants health (level of health in migrant communities, health care access) | None has been explicitly publicised. However, the Ministry of Health has defined in 2005 four health problem-areas on which more emphasis should be put (in terms of epidemiology and health care, not necessarily R&D), namely, cancer, cardiovascular diseases, age-related health problems, and AIDS. For each one of these areas a national co-ordinator has been appointed. Also, a specific amount of money was allocated to research in these major areas and also looking at the implementation of the National Health Plan |
| **Slovakia** | **Ministry of Health** | **Ministry of Education** |
|  | Very broad, not specifying any particular field. Priorities for Slovakia are the exposure to POPs, some toxic metals, particulate matter in the air, allergies and atopia in children | - |
| **Spain** | **Ministry of Health and Consumption** | **Ministry of Education and Science** |
|  | National Subprograme of Epidemiology, Public Health and Health Services Research. Call 2006 Specific Priorities:  1- Epidemiology of neurodegenerative, brain and vascular diseases, epilepsies, mental retard, mental illness, addiction and child learning disorders  2- Environment determinant of pulmonary inflammation and occupational disorders  3- Osteomuscular and connective tissue diseases: epidemiological studies and clinical practice assessment  4- Gender and Health  5- Obesity prevention (based on diet, physical exercise, environmental factors); Epidemiology of child obesity  6- Rare diseases: natural history, epidemiology and biogeography, population registers  7- Modelling  8- Semantic web in health  9- Primary care, health care staging, demand patterns,  10- Epidemiology surveillance, nutrition, environmental and occupational health and global health diagnosis  11- Health services research, information systems, health systems, community intervention, and health care assessment.  12- Decision analysis, health care worker –patient relationship,  13- Health Education and Promotion,  14- Health Technology assessment | **-** |
| **Sweden** | **Ministry of Health and Social Affairs** | **Ministry of Education, Research and Culture** |
|  | The objectives of the Swedish National Institute for Public Health:   - Participation and influence in society - Economic and social security - Secure and favourable conditions during childhood and adolescence - Healthier working life - Healthy and safe environments and products - Health and medical care that more actively promotes good health - Effective protection against communicable diseases - Safe sexuality and good reproductive health - Increased physical activity - Good eating habits and safe food - Reduced use of tobacco and alcohol, a society free from illicit drugs and doping and a reduction in the harmful effects of excessive gambling. | Questions related to ageing, the growth of diabetes, the relation between nutrition, physical activity and health, environmental health |
| **Switzerland** | **Federal Office for Public Health** | **State Secretariat for Education and Research** |
|  | Research priorities 2004-2007. Health politics: legislation; ethics, gender; health system; health statistics; health policy; social security; ageing (and future: innovation, migration, quality). Lifestyles: drugs, alcohol, tobacco; nutrition; cancer, environment; work, exercise (and future: mental health, muscular-skeletal, child development). Risks: infectious diseases, pharmaceuticals safety. | Current national research programmes in public health: antibiotic Resistance; endocrine Disruptors; Chronic Pain; Non-Ionizing Radiation; Genetically Modified Plants  Scientific Co-operation between Eastern Europe and Switzerland (SCOPES)  Environmental Science and Technology in Romania (ESTROM)  Cohort Studies; biobanks and data bases |
